# Inter and intra-tumor heterogeneity of paediatric type diffuse high-grade gliomas revealed by single-cell mass cytometry

**DOI:** 10.3389/fonc.2022.1016343

**Published:** 2022-12-08

**Authors:** Lucia Lisa Petrilli, Claudia Fuoco, Alessandro Palma, Luca Pasquini, Giulia Pericoli, Yura Grabovska, Alan Mackay, Sabrina Rossi, Angel M. Carcaboso, Andrea Carai, Angela Mastronuzzi, Chris Jones, Gianni Cesareni, Franco Locatelli, Maria Vinci

**Affiliations:** ^1^ Department of Onco-hematology, Gene and Cell Therapy, Bambino Gesù Children’s Hospital– IRCCS, Rome, Italy; ^2^ Department of Biology, University of Rome “Tor Vergata”, Rome, Italy; ^3^ Core Facilities, Istituto Superiore di Sanità, Rome, Italy; ^4^ Division of Molecular Pathology, Institute of Cancer Research, Sutton, United Kingdom; ^5^ Department of Laboratories-Pathology Unit, Bambino Gesù Children’s Hospital-IRCCS, Rome, Italy; ^6^ Pediatric Hematology and Oncology, Hospital Sant Joan de Deu, Institut de Recerca Sant Joan de Deu, Barcelona, Spain; ^7^ Department of Neuroscience and Neurorehabilitation, Bambino Gesù Children’s Hospital -IRCCS, Rome, Italy; ^8^ Neuro-oncology Unit, Department of Onco-haematology, Gene and Cell Therapy, Bambino Gesù Children’s Hospital-IRCCS, Rome, Italy

**Keywords:** paediatric-type diffuse high-grade gliomas (PDHGG), DMG, GBM, DIPG, single-cell, mass cytometry, heterogeneity

## Abstract

Paediatric-type diffuse high-grade gliomas (PDHGG) are aggressive tumors affecting children and young adults, with no effective treatment. These highly heterogeneous malignancies arise in different sites of the Central Nervous System (CNS), carrying distinctive molecular alterations and clinical outcomes (inter-tumor heterogeneity). Moreover, deep cellular and molecular profiling studies highlighted the coexistence of genetically and phenotypically different subpopulations within the same tumor mass (intra-tumor heterogeneity). Despite the recent advances made in the field, the marked heterogeneity of PDHGGs still impedes the development of effective targeted therapies and the identification of suitable biomarkers. In order to fill the existing gap, we used mass cytometry to dissect PDHGG inter- and intra-heterogeneity. This is one of the most advanced technologies of the “-omics” era that, using antibodies conjugated to heavy metals, allows the simultaneous measurement of more than 40 markers at single-cell level. To this end, we analyzed eight PDHGG patient-derived cell lines from different locational and molecular subgroups. By using a panel of 15 antibodies, directly conjugated to metals or specifically customized to detect important histone variants, significant differences were highlighted in the expression of the considered antigens. The single-cell multiparametric approach realized has deepened our understanding of PDHGG, confirming a high degree of intra- and inter-tumoral heterogeneity and identifying some antigens that could represent useful biomarkers for the specific PDHGG locational or molecular subgroups.

## 1 Introduction

Paediatric-type diffuse high-grade gliomas (PDHGG) are still virtually uncurable brain malignancies that affect children and young adults (1–6). Despite the recent efforts, the scientific advances made so far have not yet been translated into better patient outcome and the overall survival for most PDHGG patients is less than 15 months. The standard treatment still consists of surgical resection (whenever possible), radiation and chemotherapy (6–10). PDHGG can arise anywhere within the Central Nervous System (CNS) but about half of the lesions occur in midline locations. Since they are highly diffuse and infiltrative at diagnosis, they are impossible to resect (6, 11). In particular, the lesions affecting the pons are associated with the worst prognosis (1) and are not amenable to surgery given the critical role of the pons in controlling all vital functions (9). A major challenge for the implementation of effective therapies is the highly heterogeneous nature of PDHGG, known to drive processes such as cell proliferation, survival, invasion and migration as well as resistance to therapy. From this point of view, the PDHGG heterogeneity is a key obstacle hampering the successful implementation of treatment options and the improvement of patient survival rate (12). Recent molecular profiling and meta-analysis studies have shed light on the different PDHGG molecular subgroups and their clinico-pathological features, i.e. typical locations, histopathological features, age of onset and clinical outcome (6, 13–15). In the more recent classification of CNS tumors elaborated by the World Health Organization (WHO), the designation of the specific tumor entities reflects the “diffuse” nature of PDHGG, also discriminating them according to the location and the association with unique molecular alterations (4). The diffuse midline glioma H3K27-altered (DMG-H3K27) type includes all the tumors of the midline structures of the CNS, harboring the known K27M amino acid substitution on the H3 histone variants of *H3F3A, HIST1H3B*, *HIST1H3C* and *HIST2H3C* genes. Somatic mutations of *ACVR1* (16–19) are found almost exclusively in the pontine lesions, concomitantly to H3.1K27M mutation. Other alterations in genes such as *EGFR* (mutations or amplification) in H3K27M mutants or *EZHIP* (overexpression) in H3 wild-type midline tumors have also been identified and contributed to the most recent definition of the DMG-H3K27 tumor subtype. The hemispheric lesions are largely divided into diffuse paediatric-type high-grade glioma H3 wild-type and IDH1 wild-type (DPHGG-WT) and the diffuse hemispheric glioma H3G34-mutant (DHG-H3G34) associated with the *H3F3A* G34R/V driver mutations. In addition, the infant-type hemispheric gliomas (IHG) (4, 15) identify a group of tumors specifically affecting the infant/young child population (0-4 years old) and characterized by fusion genes involving *ALK, ROS1, NTRK1/2/3, MET*. Besides the inter-tumor heterogeneity, it has been shown that PDHGGs are characterized by profound intra-tumor heterogeneity, at genomic and phenotypic level. By using whole genome and whole exome sequencing, intra-tumor heterogeneity was demonstrated in biopsy, resection and autopsy samples, including specimens collected from multiregion and longitudinal sampling (12, 20–22). Taking advantage of PDHGG patient-derived cell lines, we have demonstrated that genomic and phenotypic heterogeneity are linked and that these tumors included distinct and heterogeneous subpopulations which interact in a functional network that confers a more aggressive phenotype and resistance to treatment, thus narrowing even further the therapeutic options for these diseases (12). In light of these considerations, there is an urgent need to fully characterize PDHGG tumor heterogeneity. The understanding of the specific cell populations and their cellular states contributing to tumor behavior, progression and response to therapy, may path the way toward future therapeutic strategies for patients with PDHGG. Such a challenging goal can be realized by exploiting the potential of a single-cell approach instead of relying on bulk tissue analysis as performed by most studies that failed in the attempt of adequately capturing tumor heterogeneity. With single-cell RNA sequencing approaches, we have started to gain more insights on the cellular lineage of glioma cells and on their plasticity through four main cellular states (neural-progenitor-like, oligodendrocyte-progenitor-like, astrocyte-like, and mesenchymal-like) dictated by the genetic make up and by the tumor microenvironment (23–25). Here we employ single-cell mass cytometry (cytometry by time-of-flight, CyTOF) (26, 27), a powerful tool that allows to simultaneously study the expression of multiple proteins (over than 40 targets) at single-cell level by means of antibodies linked to rare heavy metal isotopes. Compared to other single-cell modalities, this technology does not restrict the investigation at one level, but enables to define multiple cellular features such as protein expression level as well as post-translational modifications within the same experiment, providing a high-throughput marker quantification with single-cell resolution. We take advantage of single-cell mass cytometry to profile, for the first time, a panel of eight patient-derived cell lines from different locational and molecular PDHGG subgroups, to dissect their cellular heterogeneity at the protein level. The antibody panel adopted for the analysis included 15 markers, specifically set to recognize antigens expressed on the surface and in the intracellular compartments of brain and PDHGG tumor cells through the use of antibodies directly conjugated to metals or, in part, specifically customized to detect the unique histone variants. Our data revealed great phenotypic heterogeneity among the analyzed PDHGG cell lines and highlighted that the degree of plasticity, as well as the clusters of cells populating each cell line, differ from tumor to tumor. Moreover, it also allowed to identify key antigens specifically associated with particular PDHGG subgroups that were further investigated through RNA-seq and immunohistochemistry on a more extended panel of tumor samples.

## 2 Material and methods

### 2.1 Cell lines and culture conditions

The study was conducted according to the guidelines of the Declaration of Helsinki, and approved by the Institutional Ethical Committee of the Bambino Gesù Children’s Hospital (Ethical Committee Approvals N°1680/2018 and 2297/2020). Informed consent was obtained from all subjects involved in the study. PDHGG patient-derived cell lines were generated immediately after sample collection or from live cryopreserved tissue. Tumor tissue samples were obtained from seven patients at the “Ospedale Pediatrico Bambino Gesù (OPBG)” in Rome (Italy) and one patient at the “Hospital Sant Joan de Déu (HSJD)” in Barcelona (Spain). Cell cultures were established as previously described (28). Briefly, tumor samples were finely minced with a scalpel under a sterile hood. Homogenized tissue was gently enzymatically digested for 20 minutes at 37°C in a solution containing Liberase TL (Roche) and 1 U/mL DNase I (Thermofisher Scientific) diluted in 1X Phosphate Buffer Solution (PBS) (PanBiotech). The reaction was inactivated with Tumor Stem Medium (TSM) consisting of 1:1 Neurobasal-A Medium (Gibco) and DMEM/F-12 (Gibco) supplemented with 10 mM HEPES Buffer Solution (1 M, Gibco), 1X Non-Essential Amino Acid (100X, Gibco), 1X GlutaMAX-I Supplement (100X, Gibco), 1 nM Sodium Pyruvate Solution (100 nM, Gibco) and 1X Antibiotic-Antimycotic (100X, Gibco) and cell suspension was centrifuged at 1300 rpm for 5 minutes. Red blood cell lysis was performed by incubating cell suspension in a solution consisting of 1:1 ACK Lysis Buffer (Gibco) and TSM for 1 minute at room temperature (RT). The reaction was inactivated with TSM and sample was filtered with 70 μm cell strainers (Miltenyi Biotec) prior to being centrifuged for 5 minutes at RT. To initiate and expand primary stem-like cultures, minced tissue pellet was resuspended in TSM with the following supplements: 1X B-27 Supplement (50X, Gibco), 20 ng/mL human bFGF, 20 ng/mL human EGF, 10 ng/mL human PDGF-AA, 10 ng/mL human PDGF-BB (Peprotech) and 2 ng/mL heparin (Stem Cell Technologies) (TSM^+^). Live-cryopreserved tissue was gently but quickly thawed at 37°C, transferred into 10 ml of TSM^+^ and centrifuged at 1300 rpm for 5 min. The supernatant was gently removed and tissue pellet was resuspended in 1 ml of TSM^+^ and mechanically dissociated. For this study, PDHGG patient-derived cultures were initiated and expanded as adherent on laminin (10 μg/mL, Merck), on precoated tissue culture flasks. Cell cultures were routinely authenticity verified, using Short Tandem Repeat (STR) DNA fingerprinting service provided by Eurofins Genomics (**Table S1**) and tested for mycoplasma.

### 2.2 DNA extraction and sanger sequencing

DNA extraction and sanger sequencing was performed as previously described (28).

### 2.3 Immunofluorescence assay

For the immunofluorescence assay, cells were seeded onto laminin (10 μg/mL, Merck) precoated chamber slides. Once cells were subconfluent, the medium was removed and PDHGG adherent cells were washed with 1X PBS and fixed with 4% paraformaldehyde (PFA) for 10 minutes at RT. Cells were washed twice with 1X PBS, permeabilized with 0.5% Triton X-100 in 1X PBS for 10 minutes at RT and non-specific bindings were blocked with 10% Normal Goat Serum (NGS) in 1X PBS for 1 hour at RT. Incubation was performed by diluting metal-tagged primary antibodies in a solution containing 1% Bovine Serum Albumin (BSA) and 2% NGS in 1X PBS (IFF). H3K27M-175Lu (Abcam #ab190631, RRID : AB_2860570 metal conjugated antibody, 1:100) incubation was performed for 1 hour RT while H3.3G34R-170Er (RevMab, #31-1120-00, RRID : AB_2716433 metal conjugated antibody, 1:100) incubation was performed for 20 minutes at 37°C. Cells were then washed twice and incubated with Goat anti-Rabbit secondary antibody (Alexa Fluor 488, ThermoFisher) diluted in IFF for 1 hour at RT. Nuclei were counterstained with 1 mg/ml Hoechst33342 (Invitrogen) for 5 min at RT. Samples were acquired using LEICA fluorescence microscopy (DMi8).

### 2.4 Mass cytometry workflow

#### 2.4.1 Preparation of single-cell suspensions for CyTOF

For single-cell mass cytometry experiments, 3 x 10^6^ cells of each sample were used. Once removed medium and washed twice with 1X PBS w/o Calcium and Magnesium (Euroclone), adherent cells were incubated with Accutase (Carlo Erba) for 5 minutes. Detached cells were resuspended in TSM**^+^** and centrifuged at 1300 rpm for 5 minutes. Viability staining was performed by incubating cell suspensions in Rh-103 (Fluidigm), diluted 1:500 in TSM^+^ for 15 minutes at 37°C. Reaction was inactivated with TSM^+^ and cells were centrifuged for 5 minutes at 1300 rpm at RT.

#### 2.4.2 Cell barcoding

To minimize inter-sample antibody staining variation a palladium-based barcoding approach on fixed cells was applied. Cells were fixed with 1 mL of Fix I Buffer (Fluidigm) and incubated for 10 minutes at RT. The fixation was quenched by adding the Barcode Perm Buffer (Fluidigm) and the different samples were centrifuged at 800 g for 10 minutes. Samples were individually barcoded by incubating cell pellets with the appropriate combination of Palladium isotopes from the Cell-ID™ 20-Plex Pd Barcoding Plate (Fluidigm) in Barcode Perm Buffer for 30 minutes at RT. The staining was quenched with MaxPar Cell Staining Buffer (Fluidigm) and cells were centrifuged at 800 g for 10 minutes.

#### 2.4.3 Antibodies for mass cytometry and antibody staining

Most of the metal-tagged antibodies employed in the study were purchased from Fluidigm. In order to comply the absence of available conjugated antibodies targeting the specific mutated histone proteins, histone primary antibodies were bought and conjugated with metals. H3K27M antibody (Abcam #ab190631, RRID : AB_2860570) was purchased from the vendor and Magne^®^ Protein G (Promega) Purification kit was used according to manufacturer’s instructions for antibody purification prior to in-house conjugation with metal. Carrier-free antibody was then conjugated using the MaxPar X8 antibody-labeling kit (Fluidigm) according to manufacturer’s instructions. The yield of the antibody retrieved after the conjugation step was assessed by a plate reader (Synergy H1, BioTek, RRID : SCR_019748) and antibody was stored at 4°C at the concentration of 0.5 mg/ml in stabilizing solution (Candor Biosciences) supplemented with 0.05% sodium azide. H3.3G34R antibody (RevMab, #31-1120-00, RRID : AB_2716433) was purchased from the vendor in a functional grade formulation and conjugated with metal tag (170Er) by taking advantage of the MaxPar Antibody Conjugation Service (Fluidigm). For the antibody staining with metal tagged antibodies, samples were pooled into one tube and the surface antibody staining protocol was performed according to manufacturers’ instructions. After incubation for 30 min at RT, cells were washed twice with MaxPar Cell Staining Buffer (Fluidigm) and permeabilized with 100% ice cold methanol for 10 minutes on ice. Upon membrane permeabilization, cells were washed twice with MaxPar Cell Staining Buffer (Fluidigm) and incubated with antibodies against intracellular antigens for 30 minutes at RT according to manufacturers’ instructions. The full list of antibodies is detailed in **Table 1**. After intracellular antibody staining, cells were washed twice with MaxPar Cell Staining Buffer and incubated overnight at 4°C in the intercalator Iridium (191Ir-193Ir) (Fluidigm) according to manufacturer’s instructions.

**Table 1:** Summary of the 15 antibodies used for the mass cytometry analysis.

Antibody	Tag	Company	Product Identifier #	RRID
Anti-Human CD31	145Nd	Fluidigm	3145004B	AB_2737262
Anti-Human CD34	149Sm	Fluidigm	3149013B	AB_2756285
Anti-Human CD63	150Nd	Fluidigm	3150021B	
Anti-Human CD36	152Sm	Fluidigm	3152007B	AB_2802106
Anti-Human CD29	156Gd	Fluidigm	3156007B	
Anti-Human CD90	159Tb	Fluidigm	3159007B	AB_2893063
Anti-Human CD140α	160Gd	Fluidigm	3160007A	
Anti-Human CD49c	161Dy	Fluidigm	3161016B	
Anti-Human CD56	163Dy	Fluidigm	3163007B	
Anti-Human CD61	165Ho	Fluidigm	3165010B	
Anti-Cross GFAP	143Nd	Fluidigm	3143022B	
Anti-Human Nestin	151Eu	Fluidigm	3151013A	
Anti-Human Musashi-1	155Gd	Fluidigm	3155013B	
Anti-Human H3.3G34R*	170Er	RevMab	31-1120-00	AB_2716433
Anti-Human H3K27M*	175Lu	Abcam	ab190631	AB_2860570

The panel shows the antibody target, the metal isotope tag, the manufacturer company and the relative product identifier number (#). (*) Asterisks denote antibodies that were custom conjugated using either the Antibody Conjugation Service (Anti-Human H3.3G34R) or MaxPar Metal Conjugation Kit (Anti-Human H3K27M).

#### 2.4.4 Data acquisition

Before acquisition, cell suspension was washed once with MaxPar Cell Staining Buffer and twice with MaxPar Water and filtered through 30 μm filter-cap FACS tube. Cells were then resuspended at 2.5 x 10^5^ cells/mL in MaxPar Water containing 10% of EQ™ Four Element Calibration Beads (Fluidigm) and acquired on a CyTOF1 mass cytometer system (Fluidigm).

### 2.5 RNA sequencing analysis

RNAseq dataset are from Mackay et al., 2017, Mackay et al., 2018, Carvalho et al., 2020 and Izquierdo et al., 2021 (6, 10, 29, 30). Data was aligned with STAR to ensembl hg37, counted using HTSeq and normalized with rlog transformation in DESEq2. Data for cell cultures were from a total of 68 individual patients (H3.1K27M n=7; H3.3G34RV n=5; H3.3K27M n=33 and WT n=23) while data for tumors were from 133 individual patients (H3.1K27M n=5; H3.3G34R n=10; H3.3K27M n=52; WT n=66).

### 2.6 Immunohistochemistry and image analysis for GFAP expression on tumor tissue slide

Immunohistochemistry was carried out on formalin-fixed paraffin-embedded (FFPE) sections using an automated immunostainer (Dako Omnis). A primary antibody directed against GFAP (polyclonal, prediluited, high pH, DAKO) was applied. GFAP stained tissue slices were acquired using the Nanozoomer (Hamamatsu, RRID : SCR_022537) instrument. Slides were scanned at 40X and images were saved into.ndpi format and viewed using the NDPIv2 software (Hamamatsu). 3 random images at 20X magnification were exported from 11 PDHGG patient tissues (n=5 for H3.1 K27M; n=6 for H3.3 K27M) as.TIFF file and analyzed using the ImageJ software (RRID : SCR_003070, http://imagej.nih.gov/ij/) as described in Negm et al. (31). The mean intensity feature evaluated for each image was normalized over the number of manually counted nuclei for each image.

### 2.7 Quantification and statistical analysis

#### 2.7.1 Mass cytometry data normalization and gating

**Figure 1:**
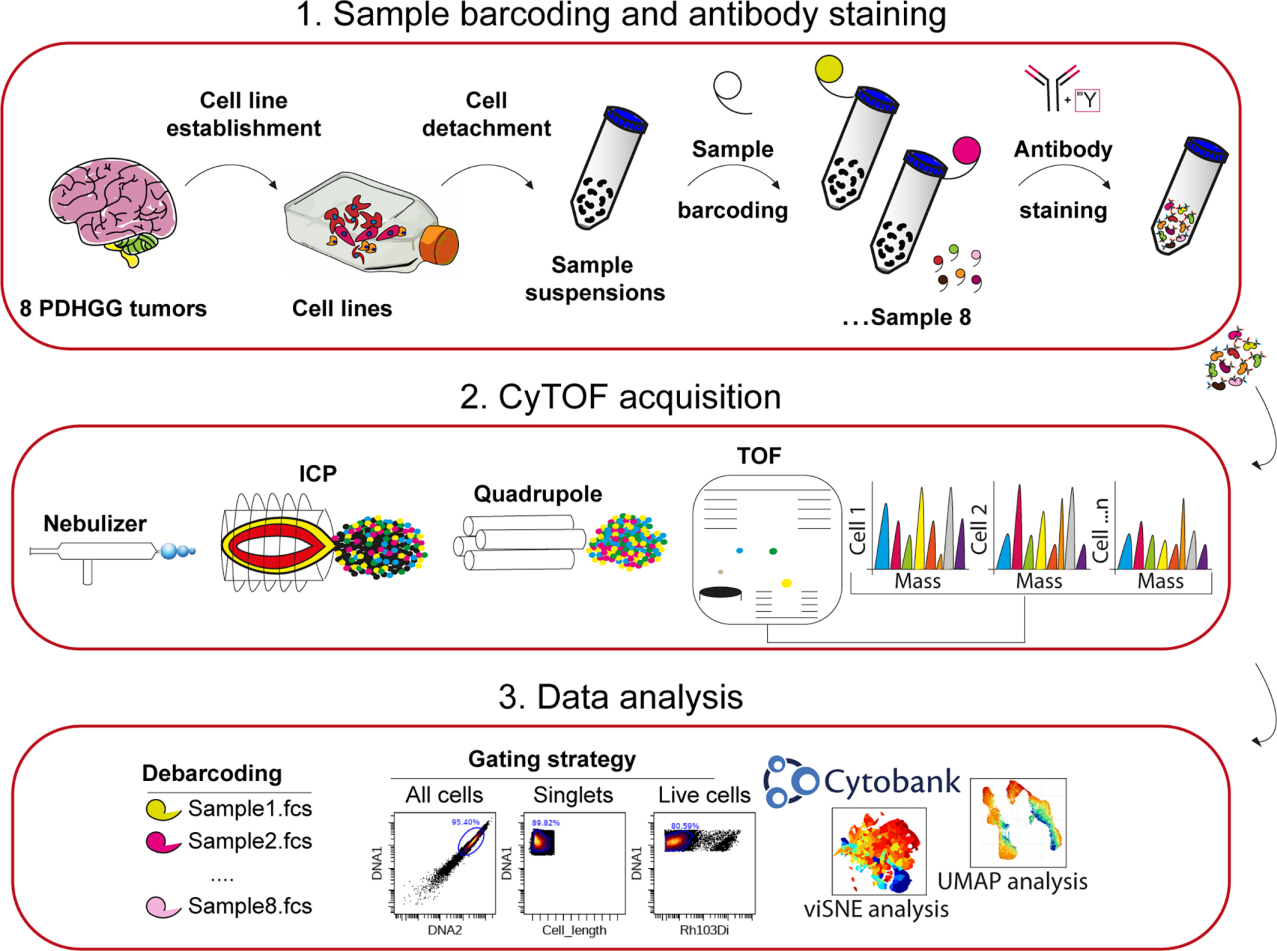
Experimental workflow adopted for single-cell mass cytometry analysis. 1. Eight PDHGG primary cell lines were established upon dissociation from tumor patients. After short expansion in stem-cell culture conditions, cells were detached for mass cytometry analysis. The different cell suspensions were barcoded with unique combinations of heavy metal tags and pooled together prior to staining with the selected metal-tag antibodies. 2. The cells were nebulized into a spray of single-cell droplets as they were introduced into the mass cytometer and atomized and ionized by the plasma (ICP). The resulting ion cloud was selected by the quadrupole for heavier reporter masses (>100 Da) which were profiled and quantified on their Time-Of-Flight (TOF). 3. Data were converted to.fcs file and further debarcoded and analyzed in the Cytobank environment, by employing the viSNE tool, and UMAP algorithm.

After the acquisition, raw data was bead-normalized using CyTOF software and cells were assigned back to their initial samples (debarcoded) by using the commercially available debarcoder software (Fluidigm). Normalized data were then uploaded onto the Cytobank (RRID : SCR_014043) environment to perform initial gating strategies (**Figure 1**). Briefly, cells were manually gated from debris on the basis of DNA content monitored by the incorporation of the Iridium (Ir) intercalator. Doublets were then excluded according to the event length parameter and single live cells were finally manually gated by using the Rhodium (Rh103) intercalator signal.

#### 2.7.2 Data visualization, analysis and accessibility

**Figure 2:**
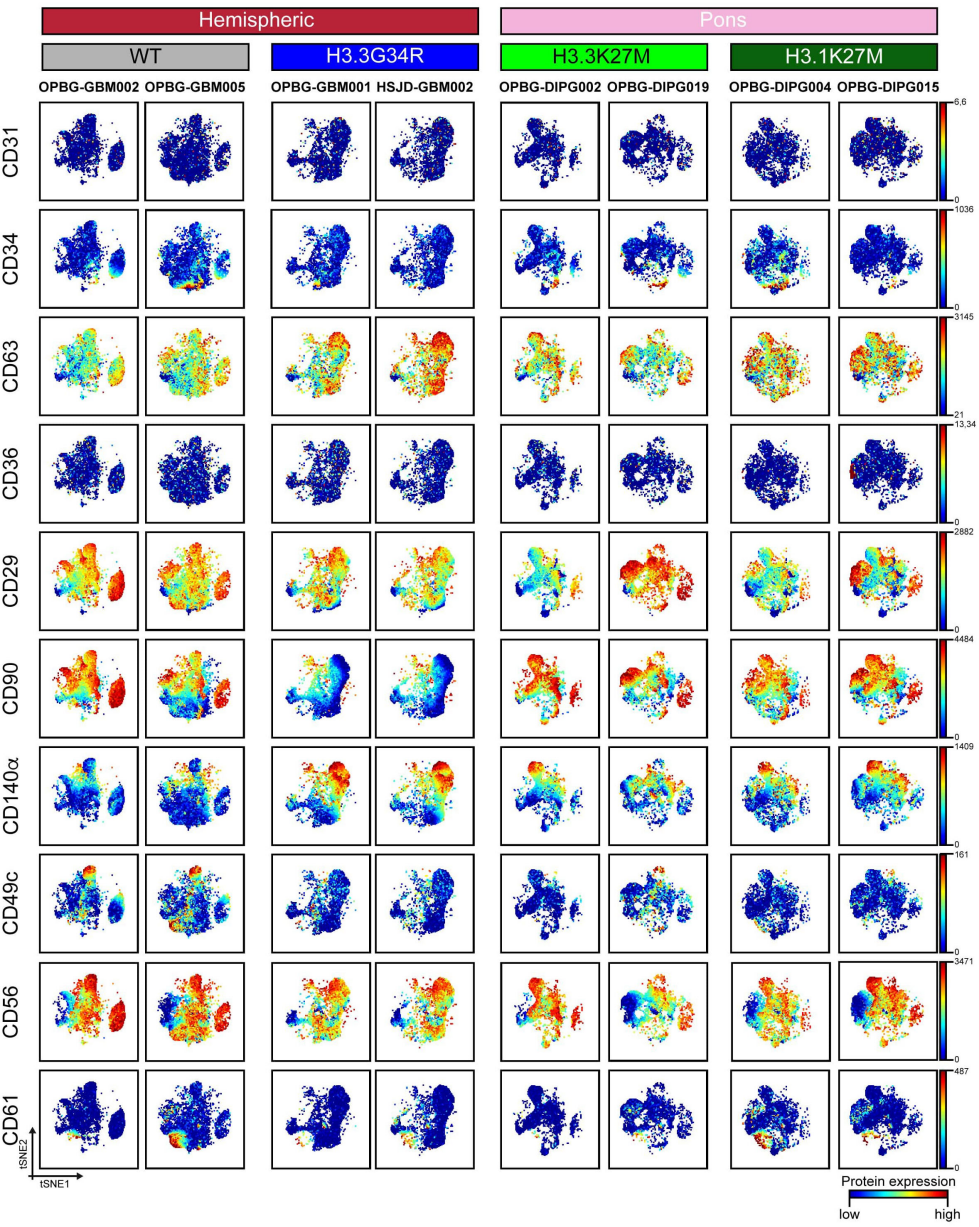
CyTOF single-cell analysis of surface antigens in PDHGG patient-derived cell lines. t-SNE maps showing the expression of 10 surface markers (CD31, CD34, CD63, CD36, CD29, CD90, CD140a, CD49c, CD56 and CD61) in each of the eight different PDHGG patient-derived cell lines analyzed through mass cytometry technique. The color gradient refers to the intensity of the expression of the considered marker, in a blue to red scale indicating low and high intensity respectively.

**Figure 3:**
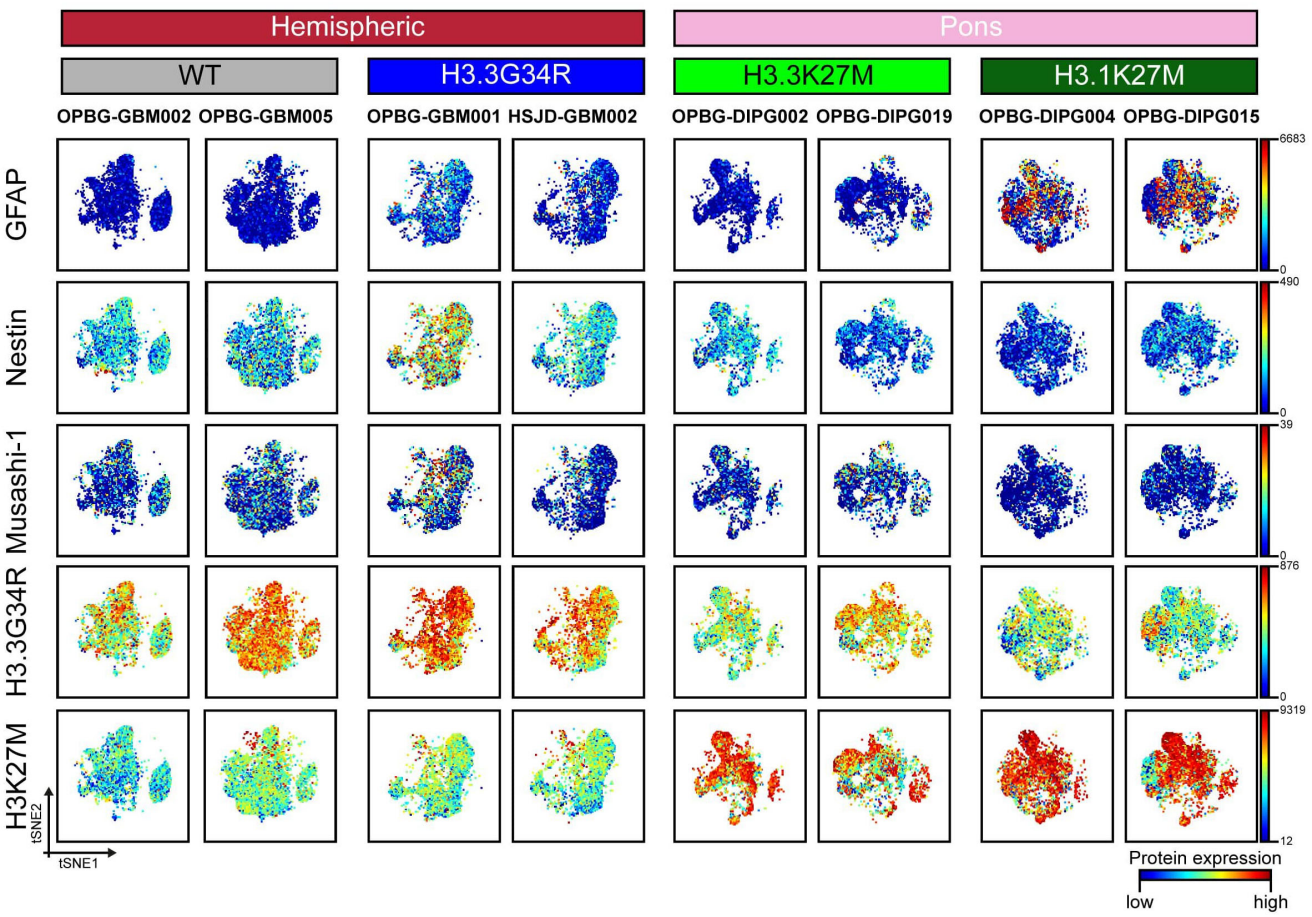
CyTOF single-cell analysis of intracellular antigen in PDHGG patient-derived cell lines. t-SNE maps showing the expression of 5 intracellular markers (GFAP, Nestin, Musashi-1, H3.3G34R and H3K27M) in each of the eight different PDHGG patient-derived cell lines analyzed through the mass cytometry technique. The color gradient refers to the intensity of the expression of the considered marker, in a blue to red scale indicating low and high intensity respectively.

**Figure 4:**
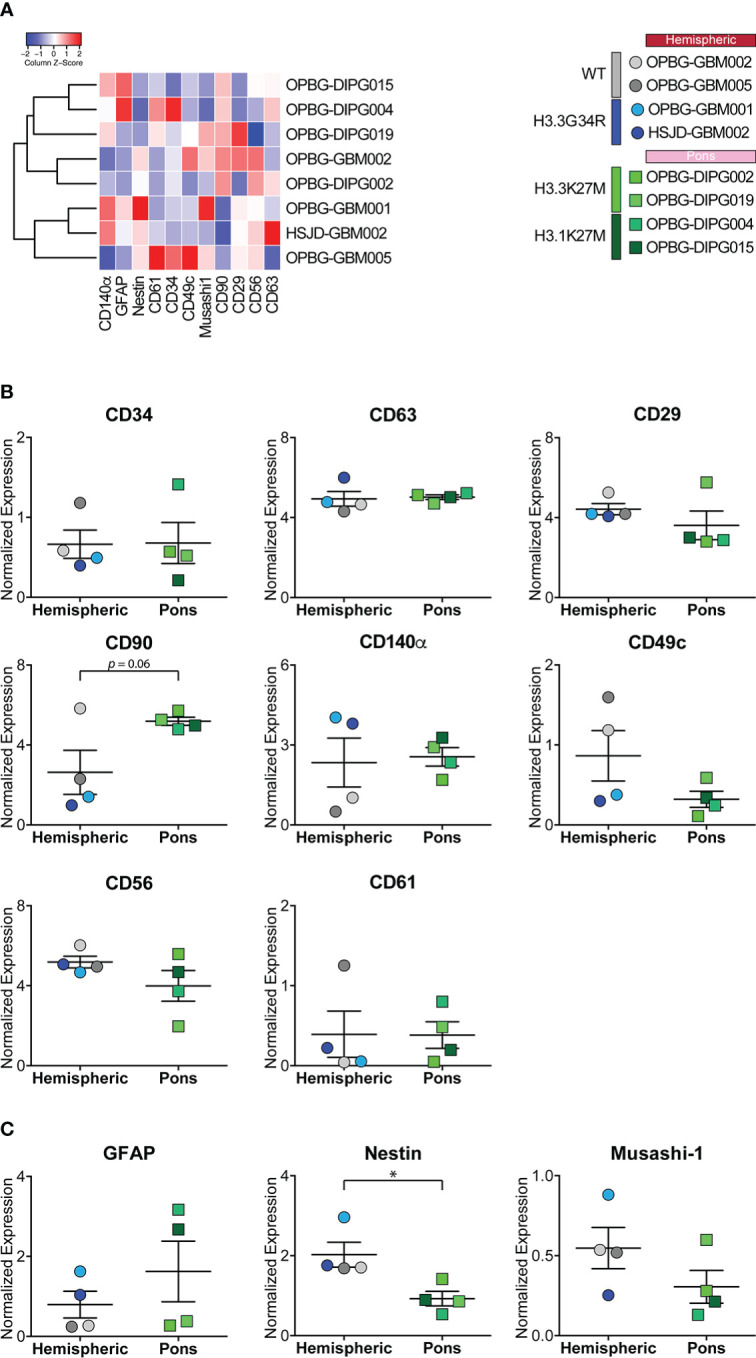
Marker expression analyzed through CyTOF technique in PDHGG patient-derived cell lines. **(A)** Heatmap summarizing the expression of the analyzed cell markers in the eight PDHGG cell lines. **(B)** Scatter dot plots showing the normalized expression of the indicated surface **(B)** and intracellular **(C)** markers in hemispheric and pontine PDHGG patient-derived cell lines. Each shape of the scatter dot plot indicates a different tumor location (round for hemispheric, square for pontine) while the color coding refers to the cell line mutational subgroups (see the key legend). *p < 0.05.

**Figure 5:**
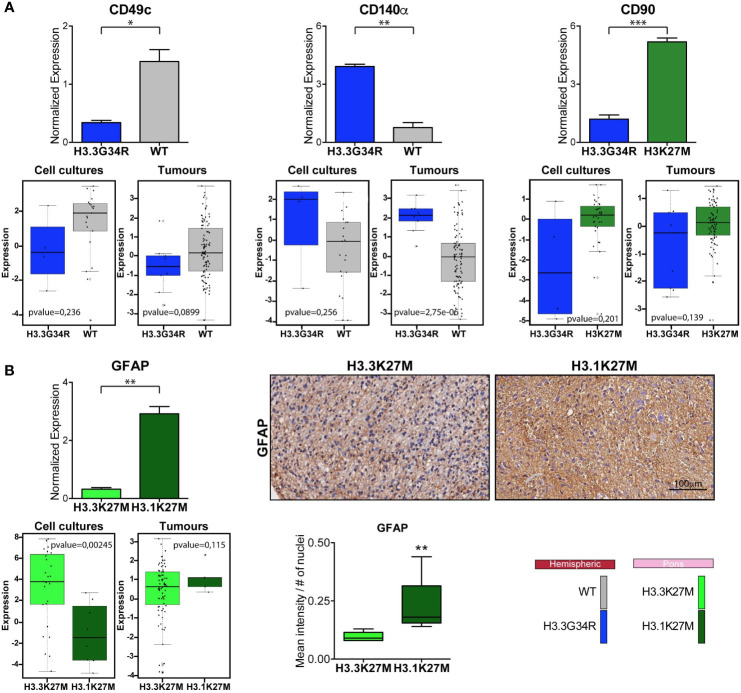
Comparison of marker expression in different molecular PDHGG patient-derived cell lines. **(A)** Bar plots showing the comparison between the H3WT *vs* H3.3G34R and H3K27M *vs* H3.3G34R PDHGG molecular subgroups relative to the expression of the indicated marker. The data on the top refer to the marker normalized expression obtained from mass cytometry data analysis while the plots on the bottom were obtained from RNA seq analysis on both patient-derived cell cultures (n=68) and tumor tissues (n=133). Data are represented as mean ± SEM. **(B)** Bar plots relative to the expression of GFAP marker in the H3.3K27M *vs* H3.1K27M PDHGG molecular subgroups obtained from CyTOF and RNA seq analysis. Representative images of GFAP immunohistochemistry on H3.3K27M and H3.1K27M PDHGG FFPE tissue slides together with the relative quantification of GFAP signal intensity normalized on the number of nuclei (n=5 for H3.1K27M; n=6 for H3.3 K27M). *p < 0.05; **p < 0.01; ***p < 0.001.

**Figure 6:**
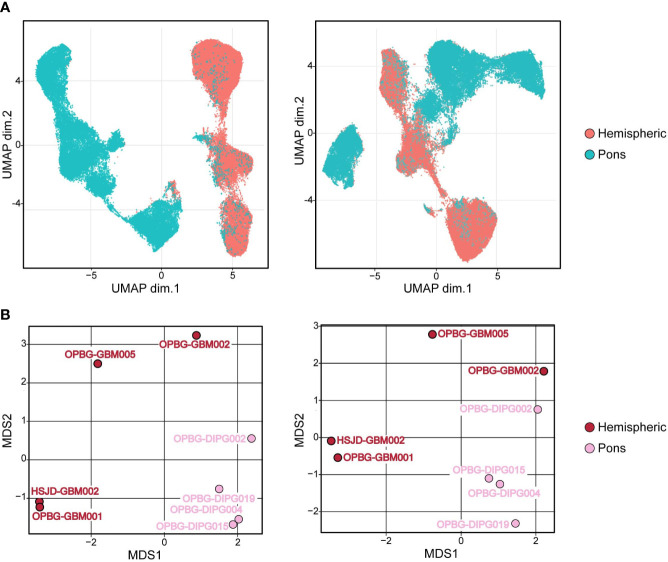
Hemispheric and pons patient-derived cell line separation. **(A)** UMAP projections and **(B)** Multidimensional Scaling plot (MDS) of PDHGG patient-derived cell lines obtained by including (left) or not (right) H3.3G34R and H3K27M histone variants in the relative analysis performed on single-cell mass cytometry data. The color refers to the locational subgroup to which the PDHGG patient-derived cell lines belong (hemispheric or pons, see the relative key legend).

**Figure 7:**
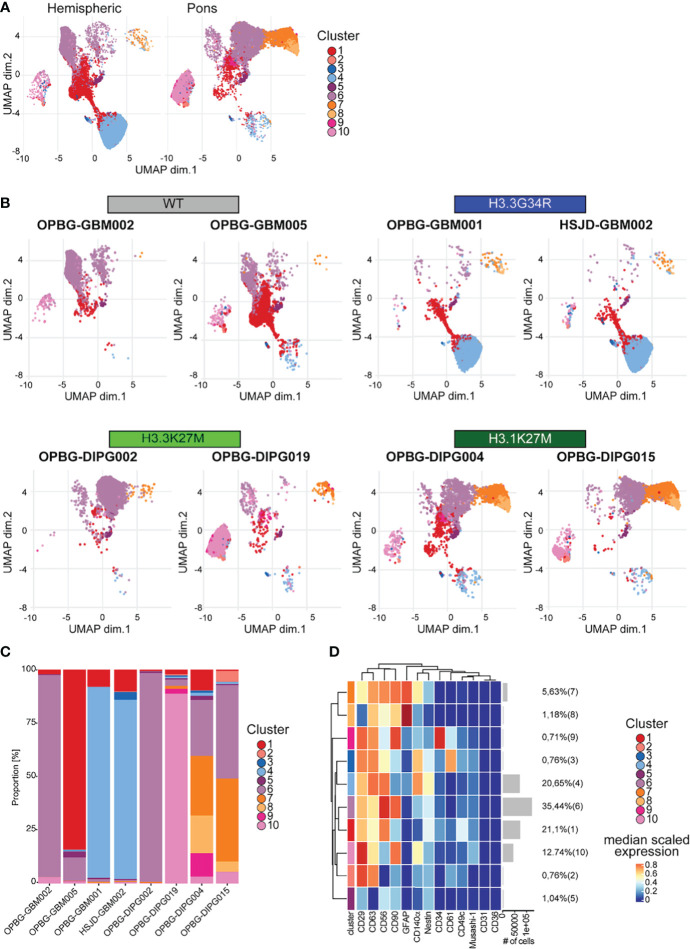
UMAP analysis. **(A)** UMAP plots of the hemispheric and pons cell lines colored according to the identified clusters. **(B)** UMAP plots for each of the analyzed cell lines, colored according to the identified cluster. **(C)** Bar plots representing the abundance of each of the clusters identified in each cell lines. **(D)** Heatmap summarizing the antigenic profile of each of the identified cluster.

For **Figures 2**, **3**, manually gated singlet (191Ir^+^ 193Ir^+^), viable (103Rh^−^) cell events were imported in Cytobank and t-distributed stochastic neighbor embedding (t-SNE) analysis was performed launching the viSNE (32) implementation in Cytobank. A proportional event sampling was selected and CD markers were chosen for clustering. The heatmap in **Figure 4A** was made with R heatmap package while marker expression in **Figures 4B, C** and **Figure 5** were derived from data processed with Cytofkit library in R environment (33), setting the following parameters: merge method: “all”, transformation method: “cytofAsinh, cluster method: “Rphenograph” with k=20, perplexity: 30, iterations: 1000, seed: 1982. UMAP analysis shown in **Figures 6A**, **7** and **Figure S3** was made using CATALYST library (RRID : SCR_017127) in R environment (34) by subsampling 1x10^4^ cells. For **Figure 7** all markers were selected for clustering and downstream analyses with the exception of H3K37M and H3.3G34R. The multidimensional scaling (MDS) illustrated in **Figure 6B** was performed on the.fcs files by using the R CATALYST (34). All statistical analyses were performed using GraphPad Prism 6.0 (GraphPad software Inc., San Diego, CA, USA, RRID : SCR_000306). Scatter dot plots show mean values ± SEM. Box plots show minimum to maximum values. Statistical significance was evaluated by the *t* test. Figures were prepared in Illustrator (Adobe, RRID : SCR_010279). Mass cytometry data have been deposited in the ZENODO open repository (https://zenodo.org/), developed under the European OpenAIRE program and operated by CERN (DOI: https://doi.org/10.5281/zenodo.7310971).

## 3 Results

### 3.1 Development and validation of a CyTOF antibody panel for PDHGG

The antibody panel was designed to include 15 antibodies, selected to profile markers relevant to primary PDHGG patient-derived cells (**Tables 1**, **2**). In particular, the panel included extracellular and intracellular antigens expressed on normal brain and tumor cells (*e.g.* GFAP, CD140α, CD90), stem cells and glioma cancer stem cells (*e.g.* Nestin, Musashi-1, CD34). Integrins and adhesion molecules, particularly relevant for the highly infiltrative nature of PDHGG, were also included (*e.g.* CD29, CD61, CD49c, CD56). Moreover, we included two markers that uniquely identify the two mutations H3.3G34R and H3K27M, associated respectively with DHG-H3G34 and H3DMG-K27M mutant cells; these antibodies were custom conjugated and validated (**Figure S2**).

**Table 2:** List of the cell antigens used together with the molecular function and reported expression.

Antigen	Other Name	Function	Expression	Reference
CD31	PECAM-1	Cell Adhesion, Angiogenesis	Endothelial Cells Glioma cancer stem cells	(35)
CD34	Hematopoietic Progenitor Antigen	Cell Adhesion	Progenitor Cells Cancer Stem Cells Mesenchymal-like Cells	(36)
CD63	Tetraspanin-30	Cell Receptor; IntegrinActivation and Signaling	Cancer Exosomes	(37)
CD36	Coll-1 Receptor	Angiogenesis, Cell Receptor	Endothelial Cells Cancer stem cells	(38, 39)
CD29	Integrin β-1	Cell Receptor	Cancer Cells	(40–42)
CD90	Thy-1	Cell-Cell/Cell-Ligand Interaction	Mature Neurons Mesenchymal Cancer Stem Cells	(43, 44)
CD140α	Platelet-Derived Growth Factor; PDGFRα	RTK involved in Proliferation, Survival and Migration	Widely Expressed in Brain Cancer Cells	(36, 45, 46)
CD49c	Integrin α-3	Cell Receptor	Cancer Cells	(40)
CD56	N-CAM1	Cell Adhesion	Neural Lineage Glioma Cancer Stem Cells	(36, 47, 48)
CD61	Integrin β-3	Cell Receptor	Cancer Cells	(46, 49)
GFAP	Glial Fibrillary Acid Protein	Mechanical and Cell Strength	Astrocytes Glioma Cells	(46)
Nestin	–	Survival and Proliferation	CNS Stem Cells	(41, 46, 50, 51)
Musashi-1	RNA-binding protein	mRNA Expression Regulation	CNS Stem Cells	(51)
H3.3G34R	–	Histone Mutation	DHG-G34	(52)
H3K27M	–	Histone Mutation	DMG-K27, pilocytic astrocytoma, and glioneuronal tumors	(53)

### 3.2 CyTOF profiling of PDHGG patient-derived cell lines

To gain insight into PDHGG tumor heterogeneity through a single-cell mass cytometry approach, eight patient-derived cell lines, were established from fresh tumor tissue specimens collected through biopsy and resection procedures (**Table 3**) and grown adherent on laminin. The cell lines were derived from hemispheric and pontine tumors and included the main molecular subgroups: two PDHGG-WT, two DHG-H3G34 and four DMG-H3K27, of which there were two H3.3K27M and two H3.1K27M.

**Table 3:** Summary of the clinico-pathological data for the eight PDHGG primary patient-derived cell lines used for the study.

Patient cell line	Gender	Age (years)		Procedure	Diagnosis	Location	Mutation	
OPBG-GBM002	M	11	Resection		PDHGG-WT	Hemispheric	WT
OPBG-GBM005	M	9	Resection		PDHGG-WT	Hemispheric	WT
OPBG-GBM001	M	12	Resection		DHG-H3G34	Hemispheric	H3.3G34R
HSJD-GBM002	M	14	Biopsy		DHG-H3G34	Hemispheric	H3.3G34R
OPBG-DIPG002	F	6	Biopsy		DMG-H3K27	Pons	H3.3K27M
OPBG-DIPG019	M	8	Biopsy		DMG-H3K27	Pons	H3.3K27M
OPBG-DIPG004	M	6	Biopsy		DMG-H3K27	Pons	H3.1K27M
OPBG-DIPG015	F	4	Biopsy		DMG-H3K27	Pons	H3.1K27M	

The panel shows the information regarding the patients’ gender and age, as well as the type of procedure, diagnosis, tumor location and mutation status.

For mass cytometry analysis, primary cells were detached and the single-cell suspensions were barcoded, pooled together in a unique tube and stained with the panel of 15 metal-tag antibodies (**Table 1**) against surface and intracellular antigens (**Figure 1**). The stained single-cell suspension was analyzed with a CyTOF1 mass cytometer instrument and, after signal debarcoding, single-cell data were analyzed by applying the t-Distributed Stochastic Neighbor Embedding (t-SNE), the algorithm implemented in Cytobank, and UMAP (**Figure 1**) (54).

### 3.3 Multiparametric profiling of PDHGG patient-derived cell lines

To visualize the heterogeneity of PDHGG patient-derived cell lines, we generated two-dimensional graphs using tSNE algorithm in Cytobank which is used to analyze the protein expression at single-cell level. All the considered patient-derived cell lines were negative for the expression of angiogenic cell markers such as CD31 and CD36, which have been previously found also on glioma cancer stem cells (35, 38, 39, 46) (**Figure 2**). For other markers tested in the study, such as the cell-adhesion molecule CD56, the integrin β-1 CD29 and its activator CD63, the tyrosine-kinase CD140α and the cell membrane molecule CD90, a variable degree of antigen expression was observed in each individual cell line. In fact, when individually considering each patient-derived cell line, the expression of the abovementioned markers was highly heterogeneous, ranging, for each marker, from a low expression level, indicated in blue, to a high expression level shown in red (**Figure 2**). This intra-tumor heterogeneity was more evident for some markers such as the cell adhesion molecule CD34 and the α3 and β1 integrins (CD49c and CD61 respectively) which were expressed only by highly restricted subpopulations of some PDHGG patient-derived cell lines. For example, CD49c resulted to be specifically expressed by a group of cells whose presence was particularly highlighted in histone wild-type patient-derived cell lines (**Figure 2**). This result confirms the notion of the existence of intra-tumor heterogeneity for surface marker expression within PDHGG patient-derived cell lines.

We then looked at the expression of specific markers which are predicted to be differentially expressed by our model and tumor subtypes and that includes the two histone variants (H3K27M and H3.3G34R) as well as some stem and differentiation markers (**Figure 3**). Musashi-1 was hardly detected (maximum detection at 39) across all the tested cell lines while Nestin was diffusely expressed in the hemispheric patient-derived cell lines and, in particular, in the H3.3G34R cell line, OPBG-GBM001 (**Figure 3**). A noteworthy observation was highlighted for the glial differentiation marker GFAP, which resulted to be highly caught (maximum detection at 6683) in our conditions but exclusively in the two H3.1K27M patient-derived cell lines. Our analysis of intracellular antigens was enriched with specific custom conjugated antibodies targeting the histone mutants H3K27M and H3.3G34R which are useful to identify the specific patient-derived cell lines affected by these mutations, and to confirm, at the protein level, the histone molecular status also defined by Sanger sequencing analysis (**Figure S1**). However, while H3K27M antibody was highly specific, only targeting H3.3 and H3.1 histone mutant cells, H3.3G34R antibody was not so exclusive when used for CyTOF analysis, binding all the patient-derived cell lines regardless of the molecular subgroup to which they belong (**Figure S2**). This was particularly observed in the case of wild-type cell lines, for which a non-specific expression of the H3.3G34R antigen was observed.

As anticipated above, by looking at the overall expression of the surface and intracellular antigens targeted by our antibody panel, we could observe that some of the PDHGG patient-derived cell lines were particularly enriched in the expression of specific antigens in comparison to other cell lines included in the analysis. In order to investigate the level of inter-tumor heterogeneity by single-cell mass cytometry, we considered different subgroups of PDHGG patient-derived cell lines on the basis of their tumor location (hemispheric *versus* pontine) and their histone status (WT, H3.3G34R, H3.3K37M and H3.1K27M) and measured the expression of the surface and intracellular markers (**Figure 4**). By doing so, we were not able to define a specific pattern of expression for some markers, such as CD63, CD61 and CD34 whose expression tended to be similar in the defined subgroups. However, some relevant differences emerged between the subgroups. We highlighted that the neural and glioma cancer stem cell marker CD56 (36, 47, 48) and the integrin β1, CD29 (40–42) were more uniformly expressed within the hemispheric subgroup, regardless of their histone alterations (**Figures 4A, B**). On the contrary, two of the analyzed markers resulted to be associated with a specific histone alteration occurring in the hemispheric subgroup. For one of these, the integrin-α3, CD49c (40), we noticed a higher expression in the two histone wild-type cell lines compared to the other molecular/locational subgroups (**Figures 4A, B**). For the CD140α marker, also known as PDGFRα, which is widely expressed in the brain but often amplified and/or overexpressed in brain cancer cell lines (36, 45, 46), our analysis showed that it was specifically associated with the H3.3G34R histone alteration of the hemispheric subgroup (**Figures 4A, B**). Moreover, we observed that the mesenchymal marker CD90 (43, 44) was homogenously expressed by the pontine PDHGG patient-derived cell lines subgroup and its expression resulted to be higher especially when compared to the hemispheric H3.3G34R patient-derived cell lines. In addition, the expression level of the astroglial differentiation marker GFAP was clearly higher in the H3.1K27M histone mutant PDHGG patient-derived cell lines.

Next, we focused on the markers for which a clear pattern of expression was observed between specific locational/molecular subgroups and looked at the gene expression level for these makers on a wider panel of patient-derived cell cultures (n=68) and tumor tissues (n=133) profiled by bulk sequencing (**Figure 5**). CD49c, which, on single-cell mass cytometry data was more highly expressed in hemispheric H3WT patient-derived cell lines, and CD140α which on the contrary was specifically associated with H3.3G34R, appear to have the same trend also on RNA expression level for both primary-derived cell cultures and tumor tissue samples (**Figure 5A**). CD90, based on the CyTOF data is more highly expressed in H3K27-altered patient-derived cell lines compared to the H3.3G34R mutant cell lines (**Figure 5A**), appear to have a similar trend at the RNA expression level. While GFAP was more strongly expressed in H3.1K27M compared to H3.3K27M mutant cell lines by CyTOF analysis, the same association was not confirmed at the RNA level (**Figure 5B**). However, the IHC staining performed on FFPE patient tissue sections, validated the mass cytometry data, showing a higher expression of GFAP at protein level in H3.1K27M tumors compared to H3.3K27M.

### 3.4 UMAP analysis on PDHGG patient-derived cell lines

Next, in order to define specific cell clusters and address the cell similarity in PDHGG patient-derived cell lines, we created a two-dimensional graph using the dimensionality reduction algorithm uniform manifold approximation and projection (UMAP). To compute UMAP, we specified all the markers listed in **Table 1**, to be considered for the algorithm, including the expression of the H3.3G34R and H3K27M mutant histones. The resulting UMAP projections showed that the cells belonging to the same locational subgroup, hemispheric and pons, clustered closer although a minimal degree of overlap between hemispheric and pons patient-derived cell lines was observed (**Figure 6A**, right panel). When including H3.3G34R and H3K27M histone alterations in the UMAP algorithm settings, the separation between hemispheric and pons patient-derived cell lines was even more clear (**Figure 6A**, left panel). These results suggest that the histone mutational status may have an impact on the determination of the cell antigenic profile but it also suggests that it is not the unique discriminating factor. In fact, by performing a multidimensional scaling (MDS) analysis we show that cells belonging to the same locational and molecular subgroup cluster quite closer already when the mutational alterations H3.3G34R and H3K27M were not taken into account for the analysis (**Figure 6B**, right panel).

In order to avoid any misinterpretation deriving from a low specificity of the H3G34R antibody, we next focused on the UMAP results that were obtained without including H3G34R and H3K27M histone variants in the analysis. The existence of 10 different clusters was described in the analyzed PDHGG cell lines and, as anticipated, they showed a minimal overlap between hemispheric and pontine subgroups as displayed on the UMAP (**Figure 7A**). Moreover, patient-derived cell lines belonging to the same mutational subgroup show a high degree of overlap, even if the expression of the mutant histones was not taken into consideration for the analysis (**Figure 7B**). Focusing on the analysis of UMAP cluster composition, the two H3.3G34R mutant cell lines were highly uniform and largely characterized by cluster 4, which was mainly distinguished by the co-expression of CD29, CD63, CD56 and CD140α (**Figures 7B–D** and **Supplementary Figure S3**). The subcomposition of the cell lines belonging to the H3.1K27M molecular subgroup was also comparable, with cluster 6 and cluster 7 being the predominant clusters for both OPBG-DIPG004 and OPBG-DIPG015 cell lines. Cluster 7 was characterized by the co-expression of GFAP at higher lever, CD90, CD63 and CD56, while cluster 6 was identified by a higher expression level of CD56 and CD90 and, at a lower level, of CD29. On the contrary, the subcomposition of the histone wild type and H3.3K27M cell lines differed between the two cell lines within each group. In fact, the OPBG-GBM002 Histone WT cell line was dominated by cluster 6, whereas OPBG-GBM005 WT cell line was enriched in cluster 1, identified by the expression of CD56 and CD29, although cluster 6 was also present. For the DMG-K27 subgroup, OPBG-DIPG002 was distinguished by cluster 6 too, while OPBG-DIPG019 was mainly dominated by cluster 10, identified by the expression of CD29, at high level, CD90 and, at a lower level, CD63 and CD140α (**Figures 7B–D** and **Supplementary Figure S3**).

## 4 Discussion

The intra and inter-tumor heterogeneity is a hallmark feature of PDHGG contributing to major failure for treatment options and resistance to therapies attempted so far (55–58). Moreover, heterogeneity has also implication on the identification of reliable biomarkers useful for the diagnostic and prognostic stratification of the patients. In fact, molecular profiling studies highlighted that PDHGG tumors could be stratified into different subgroups according to their genetic signature, driving the clinico-pathological features of these malignancies (6, 13, 14). The inter-tumor heterogeneity which distinguishes PDHGG, contributes to identify subgroups different from one to the other, explaining the failure to find a unique treatment for all. Moreover, each individual tumor mass is characterized by different cell types that are organized in a well-defined functional network, in which normal brain cell compartments are also recruited, and that, by contributing to the aggressive phenotype of PDHGG malignancies and to their resistance to therapies, undermine even further the possibility to find a more effective therapeutic strategy for these deadly diseases. To date, most studies have focused their attention on genetic heterogeneity and just a few of them have analyzed phenotypic diversity. In this study, in order to enhance our comprehension on the inter- and intra-tumor heterogeneity underlying PDHGG tumors, we take advantage of the CyTOF technology to investigate the expression of multiple extracellular and intracellular markers at single-cell resolution. To this end, we adopted a panel of 15 antibodies, designed to capture the phenotypic plasticity of PDHGG cells by targeting antigens expressed by tumor and stem-like cells as well as by normal brain microenvironment components. In particular, we characterized the single-cell phenotypes of eight patient-derived tumor cell lines, carrying different genetic alterations and arising from two distinct locational compartments of the brain, the hemispheres and the pons. By applying this approach, we obtained clear evidence that PDHGG patient-derived cell lines consisted of heterogeneous cells exhibiting dissimilar antigenic profiles, with cells expressing markers at a high level and cells completely negative for the same antigens within the same patient-derived cell line. This intra-tumor heterogeneity was evident especially when looking at the expression of some markers such as CD49c (integrin α3), CD61 (integrin β3), and CD34, which were restricted to distinct subclones. CD49c, CD61 and CD34, are all hallmarks of tumor aggressiveness: CD49c, by cooperating with EGFR, has been shown to contribute in driving tumor cell motility and invasion especially in histone WT patient-derived cell lines (59); CD61 is one of the most widely studied members of the integrin family, involved in tumor progression (60, 61) while CD34 overexpression in glioma tissues was closely associated with higher WHO grade (III + IV) (62). These observations suggest the possibility that our analysis enables the identification of more rare subclones, potentially with a more aggressive phenotype, which is also in line with what we have previously shown (12). At the inter-tumor level, according to previous observations on glioblastoma (42, 61, 63), our analysis shows that all patient-derived cell lines analyzed express the neural cell adhesion molecule CD56, the integrin β1 CD29 and its activator CD63, even if a great variability between each patient-derived cell line was observed. The mesenchymal marker CD90, a glycoprotein known to be expressed in glioblastoma and associated with an adhesion/migration gene signature and invasive tumor features (43, 44, 64), was upregulated in the pontine tumor, regardless the molecular alterations. This association was significantly observed at protein level. Interestingly, an inter-tumor heterogeneity between cell lines belonging to the same locational subgroup also arises from our study. In this regard, great differences were observed in the expression of PDGFRα (CD140α) marker which is frequently mutated/amplified in PDHGG tumors (14, 65, 66). Although there is a heterogeneity that emerges from the single-cell analysis within each cell line, our data showed a predilection of CD140α for the DHG-H3G34 subgroup, particularly when compared to the histone WT hemispheric counterpart, both at protein and RNA level. Our findings are in line with recent data pointing to the implication of PDGFRα alteration in DHG-H3G34 (67). The critical role of this marker has been demonstrated in the co-option with G34R/V, promoting malignant gliogenesis in these tumors (25). Interestingly, both of our DHG-H3G34 mutant cell lines also carry a mutation in PDGFRα. Within the DMG-K27 subgroup, the differentiation marker GFAP was more highly expressed, only at protein level, in H3.1K27M subgroup, suggesting a more pronounced astroglial differentiated phenotype for this tumor subgroup, in line with what reported also in Castel et al., 2015 (29, 53). Most of the antibodies included in the panel were already conjugated with metal. However, in order to refine our analysis by unequivocally identifying tumor cells, we specifically customized two histone variant antibodies, anti-H3.3G34R and anti-H3K27M. The antibody we have used in our study for the H3K27M mutation is commonly used in a reliable manner in routine diagnostic setting (4). Moreover, the same antibody, custom-conjugated has been used in a recently published work that, by employing the CyTOF technology, has investigated the epigenetic rearrangements due to H3K27M alteration in a panel of DMG-H3K27M mutant cell lines (68). Interestingly, Harpaz et al., have demonstrated the existence of two epigenetically distinct subpopulations in DMG-H3K27M mutant cell lines and suggest that these differences mirror the heterogeneous expression of the H3K27M oncohistone (68). While we confirmed that the antibody anti-H3K27M can be used in a robust manner, unfortunately, the H3.3G34R antibody, which was custom-conjugated and used for CyTOF analysis for the first time in this study, did not prove accurate, due to its poor specificity. In our study, this antibody lacked specificity. In fact, besides recognizing the H3.3G34R mutant cells, it also binds H3WT and, to a minor extent, H3K27M mutant cells. However, being adopted for the first time in such a study, even if its functionality in mass cytometry analysis is not optimal and would require improvements, its specificity in IHC testing has been already questioned by others who concluded that the H3.3G34R antibody is not highly predictive for the presence of G34R/V mutation and that confirmation by sequencing is mandatory (52, 69). Although the anti-H3.3G34R antibody functionality was suboptimal, when we performed the UMAP and MDS analysis, the eight patient-derived cell lines that were tested in our mass cytometry experiments clearly separated in two subgroups when histone variants antibodies were not included in the analysis and, to a great extent, also when they were included. This observation suggests the hypothesis that patient-derived cell line antigenic profiles may be largely imprinted by their molecular alterations. In order to circumvent any alteration that could affect cell clustering due to the non-specific binding of the H3.3G34R antibody to cells, we decided to remove both histone variant antibodies from the downstream analysis. Interestingly, UMAP analysis shows that the hemispheric H3G34R and the pontine H3.1 patient-derived cell lines were more homogenous than the hemispheric WT and pontine H3.3 lines in terms of cell cluster subcomposition. The hemispheric H3G34R were mainly populated by cluster 4 (CD56^+^, CD63^+^, CD140α^+^, CD29^+^, Nestin^int^) while the pontine H3.1 were mainly distinguished by cluster 6 (CD56^+^, CD90^+^, CD29^+^, CD63^int^) and 7 (GFAP^+^, CD90^+^, CD56^+^, CD63^+^, CD29^int^, CD140α^int^).

Our mass cytometry analysis on PDHGG primary patient-derived cell lines has pointed out toward potential biomarkers given by the association of specific antigens to distinct tumor subgroups. Although cell cultures are only partially recapitulating the complexity and the heterogeneity of PDHGG patient tumors, we have shown that our mass cytometry data can be validated by IHC analysis on patient tissue sample as exemplified for GFAP staining. Additional work on further validation on patient tissue sample may demonstrate the utility of the antigenic profiles we have identified on the primary cell lines by mass cytometry analysis.

Although our study is limited by a relatively small number of antibodies and also a small number of primary patient-derived cell lines, it is the first CyTOF study of this kind across the heterogeneous repertoire of the diffuse pediatric-type high-grade glioma family, and highlighted the opportunity to apply the mass cytometry technology to this complex biological context with its relevant potential and limitations.

In the future, the use of a larger panel of metal-tagged antibodies will further highlight the multidimensional potential of mass cytometry for PDHGG. This will require though a more extensive work for the *ad-hoc* customization of specific antigens of interest, for which there still is a lack of commercially available metal-conjugated antibodies, especially for brain and brain tumors. In particular, for PDHGG it will be useful to generate focused antibody panels for pathology driven biomarkers or to study specific cellular processes such as invasion/migration, and/or to focus on specific pathways in relation to potential therapeutic treatments.

We believe that the mass cytometry technology and its multidimensional analysis capability may contribute to further advance the field of PDHGG. It can be used to comprehensively characterize patient-derived models to determine how certain antigenic profiles are retained in different culture conditions (2D *vs* 3D and organoid cultures). Moreover, the use of focused metal-tagged antibody panels may be employed to study, at single-cell level, how primary patient-derived cells respond to therapeutic approaches of interest, highlighting the identification of biomarkers, allowing to follow the dynamic modulation of multiple markers and their functional states, comparing different conditions (*e.g.* pre and posttreatment) and identifying unique cell populations responsive and/or resistant to treatment. Finally, the effort to generate a custom-conjugated antibody panel for the PDHGG and the brain tumor-immune microenvironment will offer a more expanded vision on the complexity of these tumors with more advanced CyTOF based imaging mass cytometry technology for studying patient tissue samples *in situ* at single-cell level.

In conclusion, mass cytometry analysis has shown that PDHGG patient-derived cell lines are comprised by cells having different antigenic profile at both intra- and inter-tumor level. Our study opens to the possibility of employing tumor cell antigens, identified through mass cytometry analysis, as predictive biomarkers for molecular/locational PDHGG subgroup and for patient stratification.

## Data availability statement

The raw data supporting the conclusions of this article will be made available by the authors, without undue reservation.

## Ethics statement

The studies involving human participants were reviewed and approved by Institutional Ethical Committee of the Bambino Gesù Children’s Hospital. Written informed consent to participate in this study was provided by the participants’ legal guardian/next of kin.

## Author contributions

Conceptualization, LLP, CF, and MV; methodology, LLP, CF, LP, AP, GP, ALM, YG, and SR; validation, LLP; formal analysis, LLP, AP, ALM, and YG; writing—original draft preparation, LLP; writing—review and editing, LLP, CF, AP, LP, GP, ALM, YG, AC, ANM, CJ, GC, FL, and MV; supervision, MV; reagents, cases, data and/or clinical annotation: CF, SR, AMC, AC, ANM, CJ, and GC; funding acquisition, FL and MV. All authors have read and agreed to the published version of the manuscript.
