# Supplementary material for: Inter and intra-tumor heterogeneity of paediatric type diffuse high-grade gliomas revealed by single-cell mass cytometry
Source: Front Oncol. 2022 Dec 8;12:1016343. doi: 10.3389/fonc.2022.1016343 (PMC9773089; doi:10.3389/fonc.2022.1016343)
Supplement: Supplementary file 1 [file DataSheet_1.docx]

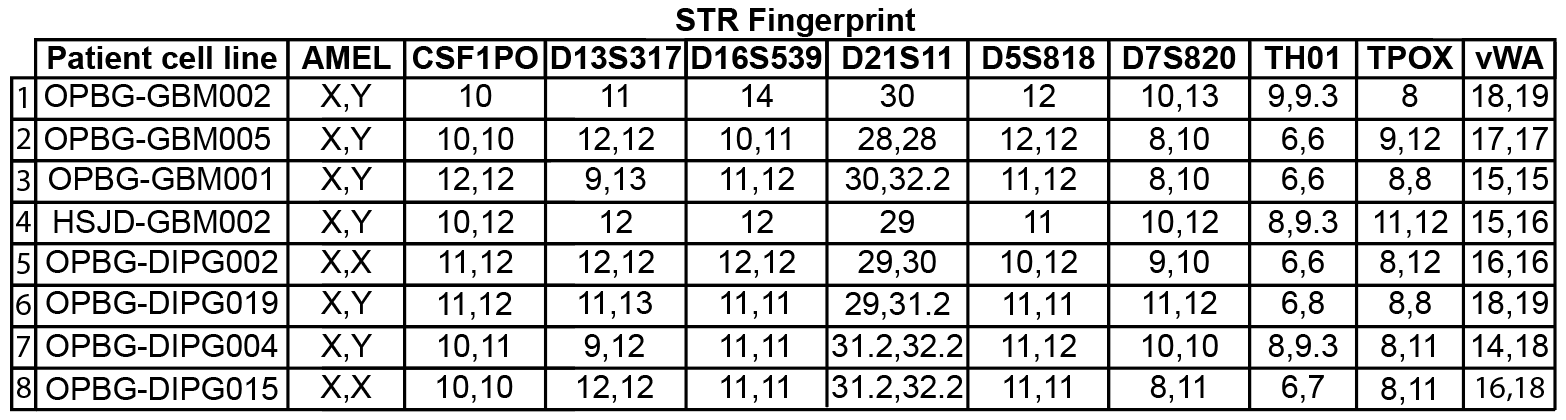


**Supplementary Table 1.** PDHGG cell line STR fingerprinting.


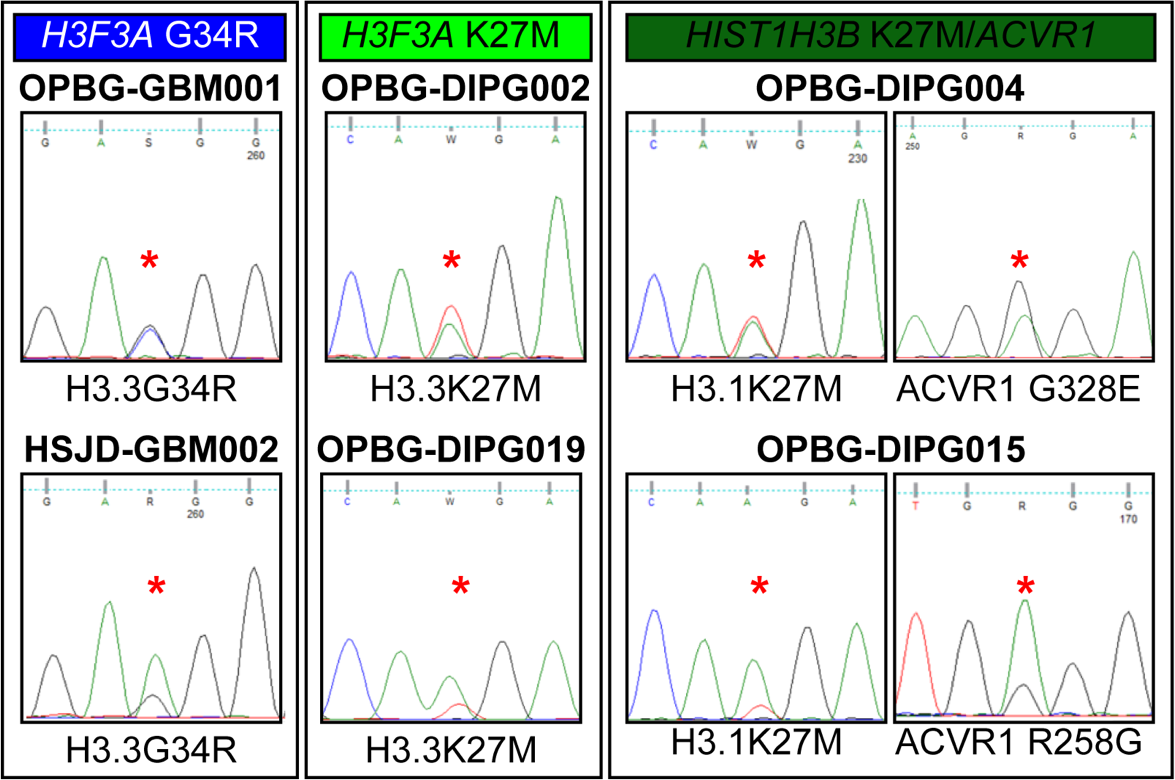


**Figure S1. Recurrent somatic mutations in histone genes.** Sanger Sequencing chromatograms showing H3F3A and HIST1H3B mutations encoding p.K27M substitutions, H3F3A mutation encoding p.G34R substitution, in the indicated patient-derived cell lines.

**
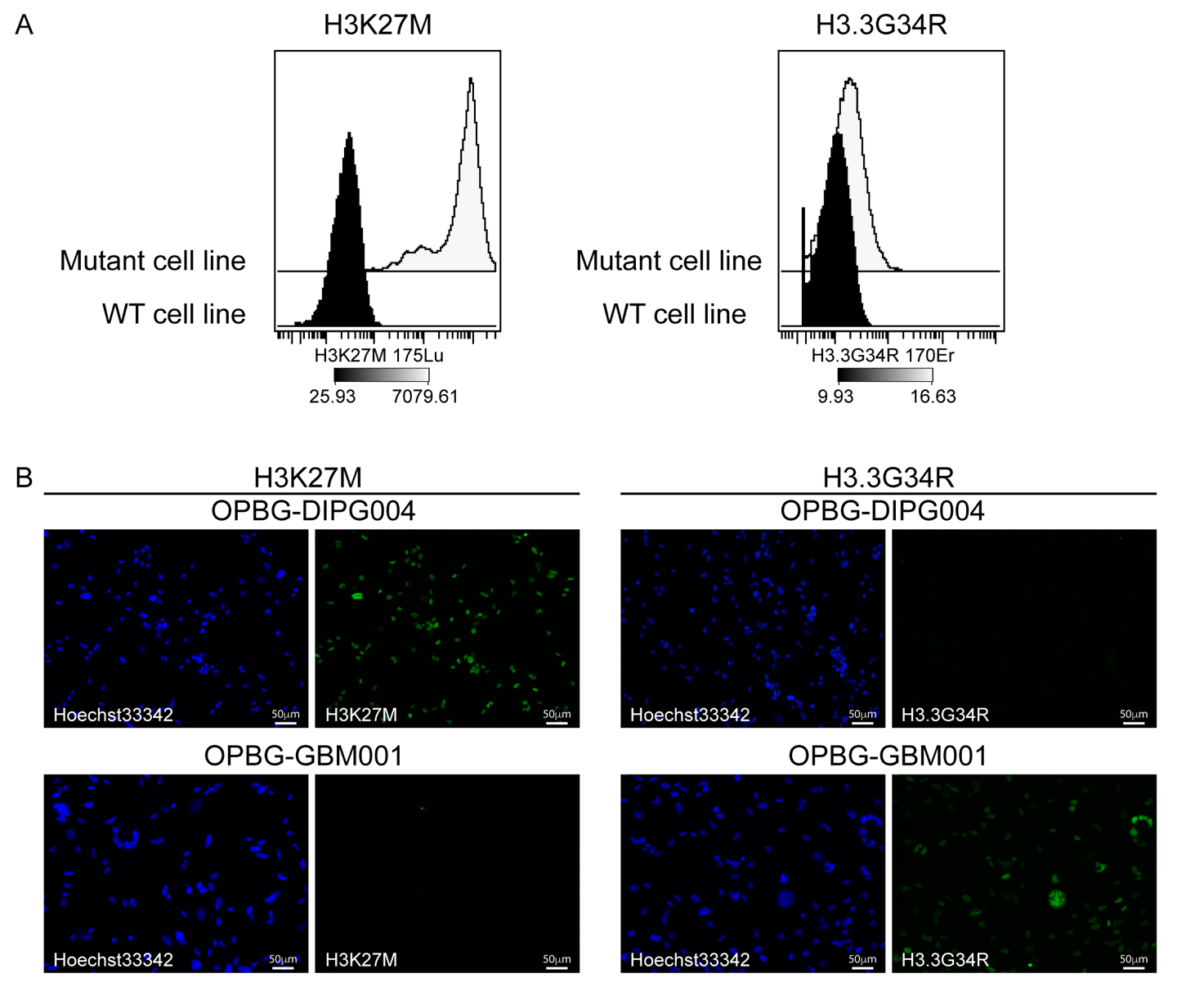
**

**Figure S2. Custom conjugated antibody validation. (A)** H3K27M and H3.3G34R custom conjugated antibodies tested by employing mass cytometry. Histograms show the overlay of the expression for each indicated marker, measured by mass cytometry, in a mutant cell line and in a wild-type cell line employed as negative control. The peaks are shaded using a color scale based on the raw values of medians for each x-axis channel. **(B)** H3K27M and H3.3G34R custom conjugated antibodies tested by employing immunofluorescence staining. H3K27M (left) and H3.3 G34R (right) staining were performed on OPBG-DIPG004 (H3.1K27M mutant) and OPBG-GBM001 (H3.3G34R mutant) patient-derived cell lines. Images were acquired using LEICA fluorescence microscopy (DMI6000B), 20x magnification 20x.


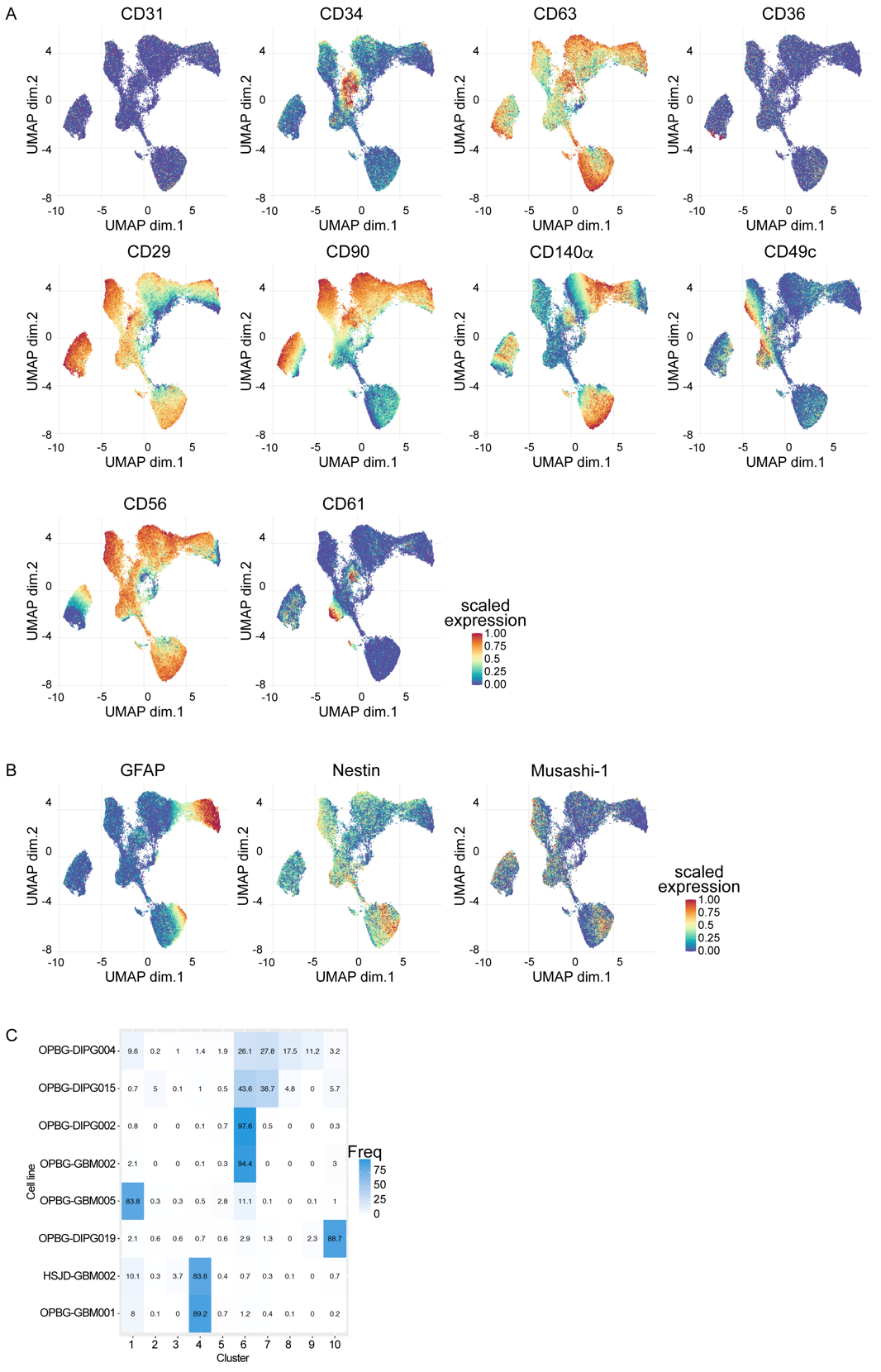


**Figure S3. Cell cluster analysis.** UMAP plots showing the expression of surface **(A)** and intracellular **(B)** markers in each of the identified clusters. **(C)** Heatmap summarizing the frequency of cluster composition for each individual cell line.
